# *Thiobacter aerophilum* sp. nov., a Thermophilic, Obligately Chemolithoautotrophic, Sulfur-Oxidizing Bacterium from a Hot Spring and Proposal of *Thiobacteraceae* fam. nov.

**DOI:** 10.3390/microorganisms12112252

**Published:** 2024-11-07

**Authors:** Anna M. Dukat, Alexander G. Elcheninov, Alexandra A. Klyukina, Andrei A. Novikov, Evgenii N. Frolov

**Affiliations:** 1 Winogradsky Institute of Microbiology, Federal Research Center of Biotechnology, Russian Academy of Sciences, 60 Let Oktjabrja Pr-t, 7, Bld. 2, 117312 Moscow, Russia; anna.dukat.m@gmail.com (A.M.D.); elcheninov.ag@gmail.com (A.G.E.); evgenii_frolov_89@mail.ru (E.N.F.); 2 Department of Physical and Colloid Chemistry, Gubkin University, Leninskiy Prospect, 65/1, 119991 Moscow, Russia; gubkin.nanotech@gmail.com

**Keywords:** *Thiobacter*, sulfur-oxidizing bacterium, aerobic respiration, chemolithoautotrophic growth, thermophilic, hot spring, Uzon Caldera

## Abstract

An aerobic, obligately chemolithoautotrophic, sulfur-oxidizing bacterium, strain AK1^T^, was isolated from a terrestrial hot spring of the Uzon Caldera, Kamchatka, Russia. The cells of the new isolate were Gram-negative motile rods with a single polar flagellum. Strain AK1^T^ grew at 37–55 °C (optimum 50 °C) with 0–1.0% NaCl (optimum 0%) and within the pH range 4.8–7.0 (optimum pH 5.2–5.5). The new isolate was able to grow by aerobic respiration with sulfide, sulfur, or thiosulfate as the electron donor and HCO_3_^−^/CO_2_ as the carbon source. The major fatty acids were C16:0, C17:1 Δ, and C16:1 ω7c. The respiratory lipoquinone was ubiquinone UQ-8. The size of the genome and genomic DNA G+C content of the strain AK1^T^ were 2.55 Mb and 64.0%, respectively. The closest 16S rRNA gene sequence of a validly published species belonged to *Thiobacter subterraneus* C55^T^ (97.94% identity). According to the 16S rRNA gene sequence-based and conserved protein sequences-based phylogenetic analyses, strain AK1^T^ represented a distinct lineage of the genus *Thiobacter* within a new family, *Thiobacteraceae* of the order *Burkholderiales*. As inferred from the morphology, physiology, chemotaxonomy, and phylogeny, strain AK1^T^ ought to be recognized as a novel species for which we propose the name *Thiobacter aerophilum* sp. nov. The type strain is AK1^T^ (=CGMCC 1.18099^T^ = UQM 41819^T^).

## 1. Introduction

The order *Burkholderiales* is phenotypically, metabolically, and ecologically diverse. This order includes strictly aerobic and facultatively anaerobic chemoorganotrophs; obligate and facultative chemolithotrophs; nitrogen-fixing organisms; and plant, animal, and human pathogens [1]. Many representatives of the order *Burkholderiales* are capable of lithotrophic growth in the presence of reduced sulfur compounds. First of all, these are facultative chemolithoheterotrophic bacteria of the genera *Limnobacter* [2,3,4,5,6], *Pollutimonas* [7,8], *Tepidimonas* [9,10,11,12], *Tepidicella* [13,14], *Comamonas* [15,16], *Caenimonas* [17], *Ottowia* [18], *Macromonas* [19], *Hydrogenophaga* [20,21,22,23], *Burkholderia* [24], and *Pandoraea* [25,26]. Moreover, chemolithoautotrophic sulfur-oxidizing representatives of the genera *Thiobacter* [27], *Thiomonas* [28,29,30,31,32,33,34,35,36,37,38,39], and *Sphaerotilus* [40,41] have been described.

The genus *Thiobacter* was described in 2005 and currently comprises the only species with a validly published name, *T. subterraneus*, which was isolated from hot subsurface aquifer water in the Hishikari gold mine, Japan [27]. *T. subterraneus* is an obligately chemolithoautotrophic, aerobic, sulfur-oxidizing bacterium growing at temperatures between 35 and 62 °C with optimum 50–55 °C and pH between 5.2 and 7.7 with optimum 6.5–7.0. Representatives of the genus *Thiobacter* were detected in Nakabusa hot springs, Japan, but their proportion in communities of microbial mats was negligible [42]. At the same time, *Thiobacter* fraction comprised up to 27% of microbial communities of subterranean thermal aquifers and terrestrial hot springs of the Karmadon Valley, Russia [43]. Thus, *Thiobacter* belongs to the physiological group of colorless sulfur bacteria and probably plays an important role in the biogeochemical cycles of carbon and sulfur in thermal habitats. Until now, the *Thiobacter* genome sequence has not been available, and subsequently, the genetic determinants of the main metabolic processes have not been identified. Moreover, the phylogenetic position of the genus *Thiobacter* at the family level within the order *Burkholderiales* remains unclear.

In this paper, we report the isolation and characterization of a new species of the genus *Thiobacter* from a hot spring of Uzon Caldera (Kamchatka, Russia), its genome analysis, and description of the genetic determinants of the main metabolic processes, focusing on the mechanisms of oxidation of reduced sulfur compounds, aerobic respiration, and pathway of CO_2_ fixation. Moreover, we report the description of a new family, *Thiobacteraceae*.

## 2. Materials and Methods

### 2.1. Sampling and Isolation

Strain AK1^T^ was isolated from a mixed sample of sediments and water collected from the hot spring of the Uzon Caldera, Kamchatka, Russia (N 54°29′56″ E 160°00′55″, elevation 664 m) in 2021. The hot spring had a circle shape about 10 cm in diameter with white-yellow filamentous fouling, under which there were black sediments (Supplementary Figure S1). The emanation of gasses in the form of bubbles rising from the hot spring bottom was observed. Conditions at the sampling site were 58 °C and pH 6.0.

The enrichment culture was obtained by the addition of 5% (*v*/*v*) of the sample to a 15 mL Hungate tube filled with 5 mL anaerobically prepared sterilized liquid medium of the following composition (g L^−1^): NH_4_Cl, 0.33; KCl, 0.33; MgCl_2_ × 2H_2_O, 0.33; CaCl_2_ × 6H_2_O, 0.33; KH_2_PO_4_, 0.33; Na_2_S_2_O_3_, 1.0; elemental sulfur, 10.0; 1.0 mL L^−1^ trace element solution [44]; 1.0 mL L^−1^ vitamin solution [45]. The head space was filled with O_2_/N_2_/CO_2_ (1:4:5, *v*/*v*). To adjust the pH of the medium to 5.5, 6 M HCl or 3 M NaOH were used. After 3 days of incubation at 50 °C, the growth of cells with various morphotypes, including numerically dominant motile rods, was observed. A serial 10-fold dilution technique under the same conditions was used to isolate a pure culture designated as AK1^T^. The purity of the strain was verified by cultivation on an organic-rich medium containing glucose and yeast extract or peptone (2 g L^−1^) at various pH (5.0–8.0) and temperatures (30–55 °C) to reveal organotrophs, as well as by analyzing the whole genome sequence assembly. All chemical reagents were manufactured by Sigma-Aldrich (Taufkirchen, Germany) according to ACS standards.

The type strain for a new taxa has been deposited in CGMCC (China General Microbiological Culture Collection Center) under a number CGMCC 1.18099^T^ and in UNIQEM Collection Core Facility (The Core Facility “Collection of Unique and Extremophilic Microorganisms”) under a number UQM 41819^T^.

### 2.2. Phenotypic Characterization

The cell morphology of the strain AK1^T^ was examined under a 1000× phase-contrast light microscope CX41RF (Olympus, Bartlett, TN, USA). Ultrathin sections of whole cells were visualized by transmission electron microscopy at the UNIQEM Collection Core Facility, Research Center of Biotechnology of the Russian Academy of Science, as described [46].

Growth experiments were performed in triplicates using Hungate tubes with a medium of the same composition as used for the pure culture isolation procedure. To determine optimal growth conditions, strain AK1^T^ was cultivated aerobically at various temperatures (7–70 °C) and pH values (4.8–7.0), adjusted with solutions of 6 M HCl or 3 M NaOH. To test the tolerance of the new isolate to NaCl concentration, it was cultivated aerobically at NaCl concentrations from 0.0 to 40.0 g L^−1^ with intervals of 5.0 g L^−1^.

Various substrates were tested as possible electron donors and carbon sources with a medium of the same composition as used for the pure culture isolation procedure but without the addition of thiosulfate and elemental sulfur. The pH value of the medium was 5.5, and the incubation temperature was +50 °C. The spectrum of tested substrates included sulfide (5 mM), thiosulfate (20 mM), elemental sulfur (10 g L^−1^), glucose, fructose, mannose, maltose, sucrose, galactose, xylose, lactose, arabinose, mannitol (5 mM), lactate, pyruvate, fumarate, acetate, citrate, oxalate, succinate, tartrate, oxalate, methanol, ethanol, propanol, isopropanol, glycerol, formate (20 mM each), yeast extract, peptone, tryptone, and casamino acids (1.0 g L^−1^). The growth of molecular hydrogen as an energy source was also examined in a medium without thiosulfate and elemental sulfur with a gas phase of 80% H_2_, 10% CO_2_, and 10% O_2_. Aerobic growth was tested with 1.0, 2.0, 5.0, 7.5, 10.0, and 20.0% oxygen in the gas phase in the presence of thiosulfate (1.0 g L^−1^) and elemental sulfur (10.0 g L^−1^) as electron donors. Anaerobic growth was tested using nitrate (20 mM), nitrite (20 mM), and fumarate (10 mM each) as electron acceptors in the presence of thiosulfate (1.0 g L^−1^) and elemental sulfur (10.0 g L^−1^). In addition, the ability for anaerobic growth was examined under the H_2_/CO_2_ gas phase (4:1, *v*/*v*) using nitrate (20 mM), nitrite (20 mM), thiosulfate (20 mM), sulfate (1.0 g L^−1^), elemental sulfur (10.0 g L^−1^), fumarate (10 mM each) as electron acceptors. Soluble substrates and electron acceptors were added from sterile anaerobic stock solutions before inoculation. Insoluble substrates and electron acceptors were added directly into the tubes with a liquid medium prior to sterilization. Utilization of substrates or electron acceptors was monitored by the increase in cell number estimated using phase-contrast microscopy and by the decrease in electron donor or acceptor concentration in the medium during growth. All chemical reagents were manufactured by Sigma-Aldrich (Taufkirchen, Germany) according to ACS standards. All experiments were performed in triplicate. Data analysis was performed using Microsoft Excel 2010 software (Microsoft, Redmond, WA, USA).

### 2.3. Analytical Methods

Sulfide was measured colorimetrically with N,N-dimethyl-*p*-phenylenediamine [47]. Sulfate, tetrationate, and thiosulfate were analyzed with a Stayer liquid chromatograph (Aquilon, Podolsk, Russia) equipped with an IonPack AS4-ASC column (Dionex, Sunnyvale, CA, USA) and conductivity detector; the eluent was bicarbonate (1.36 mM)/carbonate (1.44 mM) at a flow rate of 1.5 mL min^−1^. H_2_ and CO_2_ were analyzed with a Chromatec Crystal 5000.2 gas chromatograph (Chromatec, Yoshkar-Ola, Russia) equipped with a NaX zeolite 60/80 mesh 3 m × 2 mm column (Chromatec, Yoshkar-Ola, Russia) for H_2_ and a Hayesep Q 80/100 mesh 3 m × 2 mm column (Chromatec, Yoshkar-Ola, Russia) for CO_2_. Acetate, formate propionate, butyrate, methanol, ethanol, propanol, isopropanol, butanol, and isobutanol were assayed using a gas chromatograph with a flame ionization detector (FID), and the Optima FFAPplus 0.25 μm × 0.32 mm × 30 m capillary column (Macherey-Nagel, Allentown, PA, UK) with argon as the carrier gas. Separation was carried out with temperature programming. For gas chromatography, the samples (0.2 mL) were pre-treated by centrifugation at 12,600× *g* for 2 min, followed by acidification of clear supernatants with 5 M formic acid to pH 2.0.

### 2.4. Chemotaxonomic Analysis

Cellular fatty acids (CFA) were determined as described earlier [48] but with the CP-Sil 88 column instead of the HP-5MS column. Respiratory lipoquinones were extracted with cold acetone from cells disrupted by grinding in liquid N_2_ and further separated by TLC. The excised bands were analyzed by tandem mass spectrometry (LCQ ADVANTAGE MAX, Thermo Finnigan, San Jose, CA, USA), and the compounds were identified by their ionized masses.

### 2.5. Genome Sequencing and Assembly, Genome Annotation and Functional Genome Analysis

The genomic DNA of strain AK1^T^ was isolated from a culture grown on a Pfennig medium with thiosulfate as an electron donor, O_2_ as an electron acceptor, and CO_2_ as a carbon source. DNA extraction was performed using FastDNA™ SPIN Kit for soil (MP Biomedicals, Santa Ana, CA, USA) according to the manufacturer’s instructions, including a bead-beating stage performed on a FastPrep-24™ 5G homogenizer (MP Biomedicals, Santa Ana, CA, USA).

A shotgun WGS library preparation and sequencing were performed as described previously [49]. DNA nanoballs-based sequencing with paired-end reads was performed using the DNBSEQ-G400 platform (MGI, Wuhan, China).

After sequencing, adapters were trimmed using SOAPnuke software v.2.1.7 [50]. Further, the reads were filtered using the CLC Genomic Workbench v.10 Qiagen, Hilden, Germany), resulting in 7,856,208 pairs of high-precision reads (average length of 149.5 bp). These reads were used to assemble the genome of strain AK1^T^ in SPAdes v.3.15.4 [51] with the trusted-contigs option, for which the contigs obtained in Unicycler v.0.4.9 [52] were additionally taken. Completeness and contamination levels were estimated using CheckM v.1.2.2 with the “Bacteria” marker set [53].

The primary annotation of the genome was performed using the Prokaryotic Genome Annotation Pipeline v.6.6 [54] during the deposition of the genome into the NCBI GenBank database. Additional annotation was performed using the RAST server [55].

### 2.6. Phylogenetic Analysis

For 16S rRNA gene-based phylogenetic analysis, the 16S rRNA gene sequence of the strain AK1^T^ and sequences of type species of all genera within the order *Burkholderiales* as well as *Escherichia coli* NBRC 102203 as an outgroup were downloaded from the GenBank database. The sequences were aligned using MAFFT with the G-INS-i method [56]. The maximum likelihood phylogenetic tree was constructed in MEGA7 [57] using the GTR model (G + I, 4 categories) with 1000 bootstrap replications.

For phylogenetic analysis based on the bac120 set of conserved proteins [58], the dataset of genomes of all species within the order *Burkholderiales* (including species with *Candidatus* status) was created; low-quality genomes (completeness ≤ 50% and/or contamination ≥ 20%) were excluded from the final dataset (in a total of 851 genome assemblies). The protein marker sequences were identified in silico-translated genomes and aligned with the GTDB-tk v.2.4.0 [59]. The alignment was trimmed using the trimAL v1.4.1 with a gt option of 0.95 [60]. The phylogenetic tree was inferred in the IQ-tree v.2.3.5 [61] with LG + I + G4 model and 1000 ultrafast bootstrap replications [62]. The tree was visualized using iTOL v.6 [63].

## 3. Results and Discussion

### 3.1. Morphological and Physiological Properties

**Figure 1:**
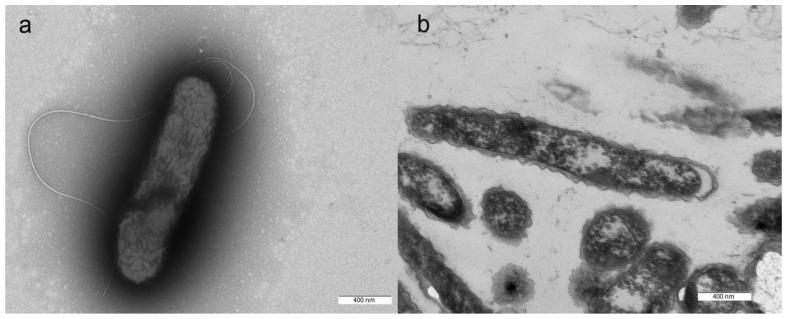
Cell morphology and ultrastructure of strain AK1^T^: (**a**) Electron micrograph showing overall cell morphology and localization of the single flagellum; bar, 400 nm. (**b**) Ultrathin section showing cell wall structure; bar, 400 nm.

Cells of the strain AK1^T^ were motile rods with a single polar flagellum, 1.0–2.0 μm in length and 0.3–0.4 μm in diameter (Figure 1a). Spore formation was not observed. Gram staining was negative. Ultrathin sections of strain AK1^T^ revealed a Gram-negative cell wall type (Figure 1b).

Strain AK1^T^ was a moderate thermophile capable of growing in the temperature range from 37 to 55 °C with an optimum at 50 °C. No growth was detected at 60 °C or above, as well as at 32 °C or below after incubation for 10 days. The pH range of growth was 4.8–7.0, with an optimum at pH 5.2–5.5. Growth was not observed at below pH 4.5 and at above pH 7.2 after incubation for 10 days. Strain AK1^T^ grew at NaCl concentrations of up to 1.0%. Optimal growth was observed on the basal medium, where the concentrations of Na^+^ and Cl^−^ were 0.1% and 0.06%, respectively. The new isolate was able to grow in both aerobic and microaerobic conditions at a concentration of oxygen in the gas phase from 1 to 20% with an optimum of 10%. The doubling time under the optimal growth conditions (50 °C, pH 5.5, no additional NaCl, 10% oxygen in the gas phase) with thiosulfate as the electron donor was 8 h.

It is important to note that among the sulfur-oxidizing bacteria of the order *Burkholderiales,* there are known moderately thermophilic species such as *T. subterraneus* [27], *Thiomonas thermosulfata* [33], *Tepidimonas* spp. [9,10,11,12], and *Tepidicella* spp. [13,14], which also prefer habitats with near-neutral pH values. Strain AK1^T^ lives in fresh water and does not tolerate high salt concentrations, while some sulfur-oxidizing representatives of order *Burkholderiales* grow at NaCl concentrations of up to 8% [7].

**Figure 2:**
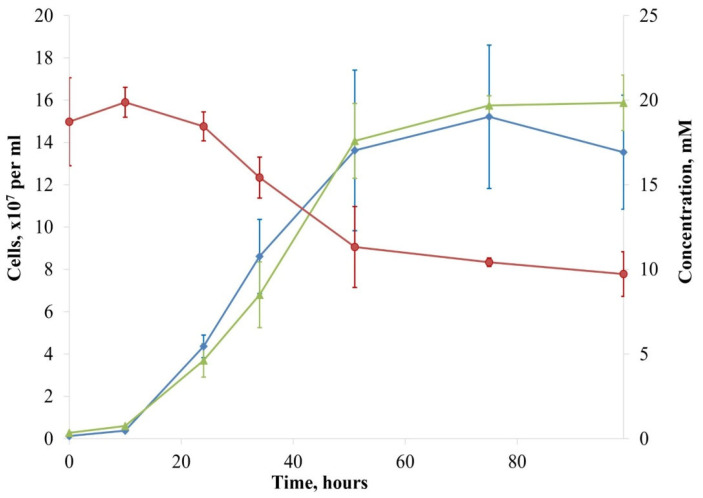
Time-courses of oxidation of thiosulfate (red), the production of sulfate (green), and concomitant bacterial growth (blue) of strain AK1^T^.

Strain AK1^T^ optimally grew chemolithoautotrophically with sulfide, sulfur, or thiosulfate as the electron donor, oxygen as the electron acceptor, and HCO_3_^−^/CO_2_ as the carbon source (Figure 2). Sulfate was the only growth product. New isolate was unable to utilize glucose, fructose, mannose, maltose, sucrose, galactose, xylose, lactose, arabinose, mannitol, lactate, pyruvate, fumarate, acetate, citrate, oxalate, succinate, tartrate, methanol, ethanol, propanol, isopropanol, glycerol, formate, yeast extract, peptone, tryptone, casamino acids, and hydrogen. Strain AK1^T^ utilized oxygen but not nitrate, nitrite, and fumarate as electron acceptors in the presence of thiosulfate and elemental sulfur. The new isolate was incapable of anaerobic growth under the H_2_/CO_2_ gas phase (4:1, *v*/*v*) with nitrate, nitrite, thiosulfate, sulfate, elemental sulfur, or fumarate as electron acceptors. Thus, strain AK1^T^, together with *T. subterraneus* [27], are the only known obligate chemolithoautotrophic aerobic sulfur-oxidizing bacteria of the order *Burkholderiales*.

### 3.2. Chemotaxonomic Analysis

The major fatty acids were C16:0 (62.4%), C17:1 Δ (15.7%), and C16:1 ω7c (14.8%). We also detected C17:0, C18:0, C22:0, and the unidentified methoxy-substituted fatty acid with an ECL = 19.22 (see Supplementary Table S1 and Supplementary Figure S2). The only quinone species present was ubiquinone UQ-8.

### 3.3. Strain Identification and Phylogenetic Analysis

**Figure 3:**
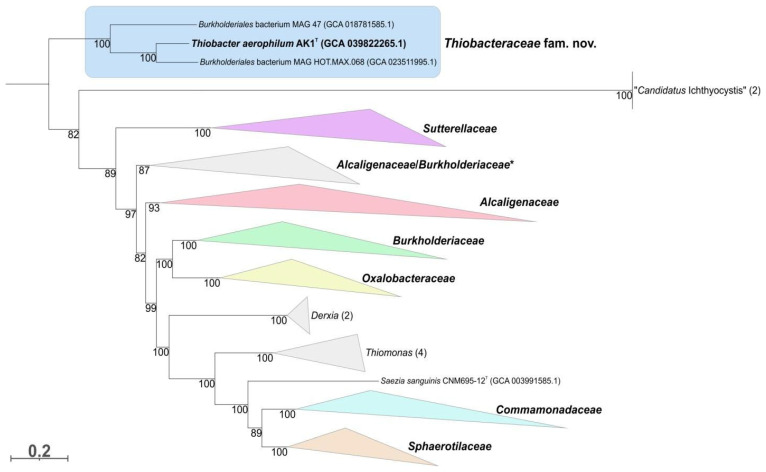
Maximum likelihood phylogenetic tree based on comparison of 120 conserved proteins and showing the position of the strain AK1^T^ (in bold) within the order *Burkholderiales*. Species are collapsed into family-level clusters (* some members from two different families formed a single cluster). The branch lengths correspond to the number of substitutions per site (see scale) according to the corrections associated with the LG + I + G4 model. The numbers at the nodes indicate the percentage of the corresponding support values. *Escherichia coli* K-12 was an outgroup.

BLAST search of homologs of strain AK1^T^ 16S rRNA gene in GenBank database revealed that strain AK1^T^ belongs to the order *Burkholderiales* with the highest sequence identity of 97.94% to the homologous gene of *Thiobacter subterraneus* C55^T^. Phylogenetic analysis based on a comparison of 16S rRNA gene sequences also placed strain AK1^T^ inside the order *Burkholderiales,* and the position of *Thiobacter* cluster was distant from known families (Supplementary Figure S3); however, support values near deep branches were low. Moreover, powerful conserved protein-based phylogenetic analysis confirmed the positioning of the clade containing strain AK1^T^ and close metagenome-assembled genomes (the genome of *Thiobacter subterraneus* is unavailable) as a separate family within the order *Burkholderiales*, which is why *Thiobacteraceae* family was proposed (Figure 3; Supplementary Figure S4). In addition, according to our analysis, some other clades (e.g., *Derxia*, *Thiomonas*, “*Candidatus* Ichthyocystis” and clusters containing *Parvibium*, *Limnobacter*, *Ephemeroptericola*, *Formosimonas,* and *Hydromonas*) could probably be reclassified in the future.

### 3.4. Functional Genome Analysis

#### 3.4.1. General Genome Properties

**Figure 4:**
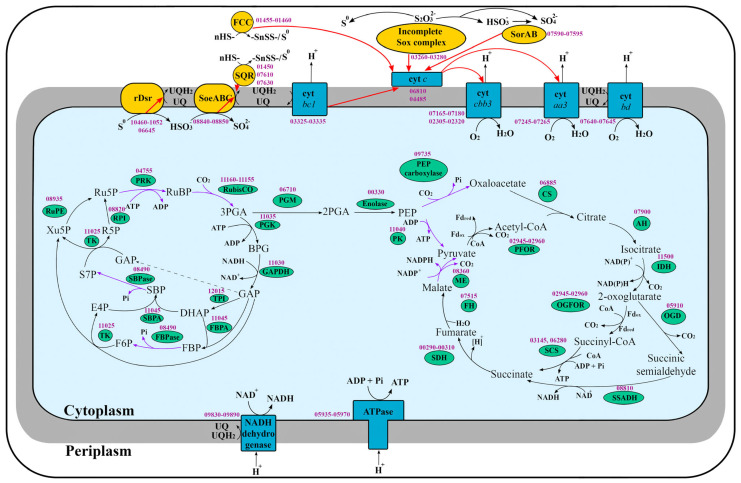
An overview of the metabolism of the strain AK1^T^ reconstructed from its genome. Abbreviations: 2PGA, 2-phosphoglycerate; 3PGA, 3-phosphoglycerate; AH, aconitate hydratase; BPG, 1,3-bisphosphoglycerate; CS, citrate synthase; cyt, cytochrome; DHAP, dihydroxyacetone phosphate; E4P, erythrose-4-phosphate; F6P, fructose-6-phosphate; FBP, fructose-1,6-bisphosphate; FBPA, fructose-1,6-bisphosphate aldolase; FBPase, fructose-1,6-bisphosphatase; FCC, flavocytochrome *c* sulfide dehydrogenase; FH, fumarate hydratase; GAP, glyceraldehyde-3-phosphate; GAPHD, glyceraldehyde-3-phosphate dehydrogenase; OGD, 2-oxoglutarate decarboxylase; OGFOR, 2-oxoglutarate:ferredoxin oxidoreductase; IDH, isocitrate dehydrogenase; ME, malic enzyme; PEP, phosphoenolpyruvate; PFOR, pyruvate:ferredoxin oxidoreductase; PGK, phosphoglycerate kinase; PGM, phosphoglycerate mutase; PK, pyruvate kinase; PRK, phosphoribulokinase; R5P, ribose-5-phosphate; rDsr, reverse dissimilatory sulfite reductase complex; RubisCO, ribulose-1,5-bisphosphate carboxylase/oxygenase; Ru5P, ribulose-5-phosphate; RuBP, ribulose-1,5-bisphosphate; RPI, ribose-5-phosphate isomerase; RuPE, ribulose-phosphate 3-epimerase; SBP, sedoheptulose-1,7-bisphosphate; SBPA, sedoheptulose-1,7-bisphosphate aldolase; SBPase, sedoheptulose-1,7-bisphosphatase; SCS, succinyl-CoA synthetase; SDH, succinate dehydrogenase; SoeABC, sulfite:quinone oxidoreductase; SorAB, sulfite: cytochrome *c* oxidoreductase; Sox complex, sulfur-oxidizing complex; SQR, sulfide:quinone oxidoreductase; SSADH, succinyl-semialdehyde dehydrogenase; TCA cycle, tricarboxylic acid cycle; TK, transketolase; TPI, triosephosphat isomerase; Xu5P, xylulose-5-phosphate.

The draft genome of the strain AK1^T^ was assembled into 32 contigs with a genome size of 2,547,564 bp and a G + C composition of 63.95%. The estimated completeness of the genome assembly was 98.28%, while contamination was 0%. When annotating the genome of the strain AK1^T^ using the RAST server, 2602 protein-coding genes and 49 RNA genes were predicted, and 2496 protein-coding genes and 52 RNA genes were predicted using NCBI PGAP. Genome analysis of the strain AK1^T^ revealed genetic determinants of the main metabolic processes (Figure 4).

#### 3.4.2. Oxidation of Reduced Sulfur Compounds

Genome analysis showed the presence of genes probably encoding three single-subunit sulfide/quinone oxidoreductases SQRs (V6E02_01450, V6E02_07610, or V6E02_07630) and one double-subunit flavocytochrome *c* sulfide dehydrogenase FCC (V6E02_01460 and V6E02_01455). These enzymes carry out hydrogen sulfide oxidation and transfer electrons to quinones or cytochrome *c*-type (V6E02_06810 or V6E02_04485). The branched Sox system, encoded by the *soxAXYZB* genes (V6E02_03260-V6E02_03280) and involved in the oxidation of thiosulfate to sulfur and sulfate, were also found in strain AK1^T^, as well as the genes *dsrABEFHСMKLJOPNRS* (V6E02_10460-V6E02_10525, V6E02_06645) of the reverse dissimilatory sulfite reductase (rDsr) complex involving in the oxidation of sulfur to sulfite. Genome analysis revealed the presence of genes for direct oxidation of sulfite to sulfate using membrane-bound sulfite/quinone oxidoreductase SoeABC (V6E02_08840, V6E02_08845, V6E02_08850) and periplasmic sulfite/cytochrome *c* oxidoreductase SorAB (V6E02_07590 and V6E02_07595). In addition, genes for indirect APS–reductase/ATP–sulfurylase pathway of oxidation of sulfite to sulfate (*aprAB*—V6E02_10375-V6E02_10370, *qmoAB*—V6E02_02625-V6E02_02620, *hdrBC*—V6E02_02610-V6E02_02615 and *sat*—V6E02_05595) were also detected.

Bacteria of the genus *Thiobacter* are the only obligate chemolithoautotrophic sulfur-oxidizing representatives of the order *Burkholderiales*. At the genome level, this is reflected in the most complete set of genes encoding sulfur-oxidizing enzymes compared to other sulfur bacteria of the order *Burkholderiales*. Strain AK1^T^ is the first representative of this order for which the presence of genes encoding sulfite/quinone oxidoreductase SoeABC and indirect APS–reductase/ATP–sulfurylase pathway of oxidation of sulfite to sulfate has been demonstrated. The presence of sulfide/quinone oxidoreductase SQR and flavocytochrome *c* sulfide dehydrogenase FCC has previously been demonstrated for *Thiomonas arsenitoxydans* [35], *Thiomonas bhubaneswarensis* [38], *Sphaerotilus sulfidivorans* [41], *Sphaerotilus montanus* [41], and *Macromonas nakdongensis* [19], while the presence of the rDsr complex and periplasmic sulfite: cytochrome *c* oxidoreductase SorAB was shown only for *Thiomonas arsenitoxydans* [35], *Thiomonas bhubaneswarensis* [38], and *Macromonas nakdongensis* [19]. All sulfur-oxidizing bacteria of the order *Burkholderiales* have genes encoding the complete Sox system (*soxABCDXYZ*) [2,3,4,5,8,10,12,19,35,38,41], except *Pandoraea thiooxydans* that oxidize thiosulfate via tetrathionate-intermediate pathway [25,26]. The complete Sox system performs complete oxidation of thiosulfate to sulfate. Surprisingly, the genome of the strain AK1^T^ contained an incomplete set of genes encoding the branched Sox system (*soxAXYZB*, without *soxCD*) that involved the oxidation of thiosulfate to sulfur and sulfate.

#### 3.4.3. Aerobic Respiration

The aerobic electron transport chain (ETC) of the strain AK1^T^ was represented by complex I NuoABCDEFGHIJKLMN (V6E02_09830-V6E02_09890, V6E02_12030), complex II or succinate dehydrogenase SdhABCD (V6E02_00290-V6E02_00310), cytochrome *bc1* of complex III (V6E02_03325-V6E02_03335), and four terminal oxidases of complex IV (cytochrome *cbb3* oxidase 1—V6E02_02305-V6E02_02320, cytochrome *cbb3* oxidase 2—V6E02_07165-V6E02_07180, cytochrome *aa3* oxidase—V6E02_07245-V6E02_07265, and cytochrome *bd* ubiquinol oxidase—V6E02_07640-V6E02_07645). During the oxidation of reduced sulfur compounds, electrons enter the aerobic ETC at the level of ubiquinones and cytochrome *c* (V6E02_06810 or V6E02_04485). Ubiquinols are oxidized by cytochrome *bc1* of complex III, after which electrons enter the cytochrome *c*. Terminal oxidases work at the final stage of aerobic respiration. The strain AK1^T^ contains four terminal oxidases, which most likely differ in oxygen affinity and operate in different ranges of oxygen concentration: two cytochrome *cbb3* oxidases have the highest affinity for oxygen and work under microaerobic conditions; cytochrome *bd* ubiquinol oxidase, which also has high affinity for oxygen and works in conditions of low oxygen concentrations; and cytochrome *aa3* oxidase, which has low affinity for oxygen and works under aerobic conditions. Such a variety of terminal oxidases with different affinities to oxygen explains the ability of strain AK1^T^ to grow in a wide range of oxygen concentrations. Interestingly, a similar situation with the presence of several types of terminal oxidases was also shown for representatives of the genus *Thiomonas*, and the authors also suggested that respiratory metabolism may occur over a wide range of oxygen tensions [35,38].

During the functioning of the ETC, a membrane potential is generated, which is partly consumed for ATP synthesis by ATP synthase (V6E02_05935-V6E02_05970) and partly for NADH production by complex I NuoABCDEFGHIJKLMN (V6E02_09830-V6E02_09890, V6E02_12030). Sulfur oxidizing organisms generate reducing power for anabolic pathways using reverse electron flow, an energy-requiring process that pushes the electrons against their thermodynamic gradient to produce NADH via complex I.

#### 3.4.4. Carbon Metabolism

We assume that CO_2_ fixation in the strain AK1^T^ proceeds via the Calvin cycle including form I ribulose-1,5-bisphosphate carboxylase/oxygenase (RubisCO) large chain (V6E02_11155), RubisCO small chain (V6E02_11160), phosphoglycerate kinase (V6E02_11035), NAD-dependent glyceraldehyde-3-phosphate dehydrogenase (V6E02_11030), triosephosphate isomerase (V6E02_12015), bifunctional fructose-/sedoheptulose-bisphosphate aldolase class II (V6E02_11045), bifunctional fructose-/sedoheptulose-bisphosphatase type I (V6E02_08490), transketolase (V6E02_11025), ribulose-phosphate 3-epimerase (V6E02_08935), ribose-5-phosphate isomerase A (V6E02_08820), and phosphoribulokinase (V6E02_04755). Assimilation of carbon dioxide via the Calvin cycle has previously been shown for both sulfur-oxidizing (*Thiomonas arsenitoxydans* [35], *Thiomonas intermedia* [39], *Sphaerotilus sulfidivorans* [41], and *Sphaerotilus montanus* [41]) and hydrogen-oxidizing (*Noviherbaspirillum autotrophicum* [64] and *Ralstonia eutropha* [65]) members of the order *Burkholderiales*.

Organic compounds from the Calvin cycle can enter the tricarboxylic acid cycle via phosphoglycerate mutase (V6E02_06710), enolase (V6E02_00330), pyruvate kinase (V6E02_11040), 2-oxoacid/ferredoxin oxidoreductases (V6E02_02945-V6E02_02960) and phosphoenolpyruvate carboxylase (V6E02_09735). We propose that 2-oxoacid/ferredoxin oxidoreductases are involved in both pyruvate oxidation and 2-oxaloglutarate oxidation. The genome of the strain AK1^T^ encodes for the tricarboxylic acid cycle including citrate synthase (V6E02_06885), aconitate hydratase (V6E02_07900), isocitrate dehydrogenase (V6E02_11500), 2-oxoacid/ferredoxin oxidoreductases (V6E02_02945-V6E02_02960), succinyl-CoA synthetase (V6E02_03145 and V6E02_06280), succinate dehydrogenase (V6E02_00295-V6E02_00310), and class II fumarate hydratase (V6E02_07515). The gene encoding NAD-dependent malate dehydrogenase was absent, while the gene encoding NADP-dependent malic enzyme (V6E02_08360) was detected. It is most likely that the tricarboxylic acid cycle is required to produce intermediates for amino acid biosynthesis.

## 4. Conclusions

Strain AK1^T^ has several features that are common with *T. subterraneus* C55^T^: it is presented by Gram-negative motile rods unable to form spores; it has one polar flagellum; it is an obligate chemolithoautotrophic aerobe oxidizing reduced sulfur compounds (Table 1). On the other hand, a number of features distinguish the strain AK1^T^ from the type species: it has a smaller cell diameter; it grows at lower temperatures and lower pH values; it grows in a much wider oxygen concentration range. Thus, strain AK1^T^ is an inhabitant of more acidic and colder environments with a higher oxygen concentration, in contrast to *T. subterraneus* C55^T^. On the basis of phylogenetic, phenotypic, and chemotaxonomic characteristics, strain AK1^T^ was identified as the type strain of a novel species within the genus *Thiobacter*, for which the name *Thiobacter aerophilum* sp. nov. is proposed.

### 4.1. Description of Thiobacteraceae fam. nov.

*Thiobacteraceae* (Thi.o.bac.ter.a.ce’ae. N.L. masc. n. *Thiobacter* type genus of the family; −*aceae*, ending to denote a family; N.L. fem. pl. n. *Thiobacteraceae* the family of the genus *Thiobacter*).

The description is the same as for the genus *Thiobacter* [27], the type genus and the sole genus in this family. The family represents a distinct monophyletic lineage as supported by phylogenetic analyses based on a comparison of conserved proteins and 16S rRNA gene sequences.

### 4.2. Description of Thiobacter aerophilum sp. nov.

*Thiobacter aerophilum* (ae.ro’phi.lum Gr. masc. n. *aer*, air; N.L. masc. adj. *philus*, loving; from Gr. masc. adj. *philos*, loving; N.L. neut. adj. *aerophilum*, air-loving, because of its ability to grow at higher oxygen concentrations in contrast to type species *T. subterraneus* C55^T^).

Cells are Gram-negative, motile by polar flagellum, rod-shaped, 0.3–0.4 µm in diameter, and 1.0–2.0 µm in length. Spore formation is not observed. Growth occurs between 37 and 55 °C, with an optimum at 50 °C. The pH range for growth is 4.8–7.0, with an optimum pH of 5.2–5.5. Growth does not occur above 1% (*w*/*v*) NaCl. Growth occurs in both aerobic and microaerobic conditions at a concentration of oxygen in the gas phase from 1 to 20%, with an optimum of 10%. It is an obligate chemolithoautotrophic aerobe. Electron donors utilized in the presence of oxygen are sulfide, thiosulfate, and sulfur. CO_2_ is the sole carbon source. No growth is observed on glucose, fructose, mannose, maltose, sucrose, galactose, xylose, lactose, arabinose, mannitol, lactate, pyruvate, fumarate, acetate, citrate, oxalate, succinate, tartrate, methanol, ethanol, propanol, isopropanol, glycerol, formate, yeast extract, peptone, tryptone, casamino acids, or hydrogen. The major cellular fatty acids are C16:0, C17:1 Δ, and C16:1 ω7c. The dominant respiratory lipoquinone is UQ-8. The G + C content of the genomic DNA of the type strain is 64.0%. The genome size is 2.55 Mb. The type strain is AK1^T^ (=CGMCC 1.18099^T^ = UQM 41819^T^), which was isolated from a hot spring of the Uzon Caldera, Kamchatka, Russia.

## Figures and Tables

**Table 1:** Comparative characteristics of strain AK1^T^ and *T. subterraneus* C55^T^.

Characteristic	Strain AK1^T^	*T. subterraneus* C55^T^
Isolation source	Terrestrial hot spring	Subsurface geothermal aquifer water
Cells	rods	rods
Cell diameter (µm)	0.3–0.4	0.4–0.5
Cell length (µm)	1.0–2.0	1.1–1.9
Flagellation	polar flagellum	polar flagellum
Temperature for growth:		
Range	37–55 °C	35–62 °C
Optimum	50 °C	50–55 °C
pH for growth:		
Range	4.8–7.0	5.2–7.7
Optimum	5.2-5.5	6.5–7.0
Oxygen for growth:		
Range	1–20%	2–10%
Optimum	10%	2–5%
Utilization of electron donors:		
Sulfide	+	+
Sulfur	+	+
Thiosulfate	+	+
Carbon source:		
CO_2_	+	+
Organic carbon	−	−
Anaerobic growth	−	−
Major cellular fatty acids	C16:0, C17:1 Δ, C16:1 ω7c	C16:0, C16:1, C18:0, iso-C18:0, C18:1
Respiratory quinone	UQ-8	ND
G + C content (mol%)	64.0	66.9
Genome size, Mb	2.55	ND

## Data Availability

The GenBank accession number for the whole genome sequence of strain AK1^T^ is JBAJEX000000000. Locus tag for the 16S rRNA gene is V6E02_12835.

## Supplementary Materials

The following supporting information can be downloaded at: https://www.mdpi.com/article/10.3390/microorganisms12112252/s1, Figure S1: Sampling site 4212, which is the isolation source of the strain AK1^T^; Figure S2: Mass spectrum of the unidentified fatty acid methyl ester with an ECL (CP-Sil 88) = 19.22; Figure S3: A phylogenetic tree based on a comparison of 16S rRNA gene sequences, constructed by the maximum likelihood method, showing the position of the AK1^T^ strain (highlighted in bold) within the *Burkholderiales* order; Figure S4: Maximum likelihood phylogenetic tree based on comparison of 120 conserved proteins and showing the position of the strain AK1^T^ within the order *Burkholderiales*; Table S1: Comparative composition of cellular fatty acids in strain AK1^T^ and its closest phylogenetic relative *Thiobacter subterraneus* C55*T*.
