# Supplementary material for: Thiobacter aerophilum sp. nov., a Thermophilic, Obligately Chemolithoautotrophic, Sulfur-Oxidizing Bacterium from a Hot Spring and Proposal of Thiobacteraceae fam. nov
Source: Microorganisms. 2024 Nov 7;12(11):2252. doi: 10.3390/microorganisms12112252 (PMC11596669; doi:10.3390/microorganisms12112252)
Supplement: Supplementary file 1 [file microorganisms-12-02252-s001.zip › microorganisms-3255525-supplementary.pdf]

Supplementary Materials to

***Thiobacter aerophilum* sp. nov., a Thermophilic, Obligately Chemolithoautotrophic, Sulfur-Oxidizing Bacterium from a Hot Spring and Proposal of *Thiobacteraceae* fam. nov.**

Anna M. Dukat, Alexander G. Elcheninov, Alexandra A. Klyukina, Andrei A. Novikov, Evgenii N. Frolov

Supplementary Table S1. Comparative composition of cellular fatty acids in strain AK1^T and its closest phylogenetic relative *Thiobacter subterraneus* C55^T. Compounds above 5% are in bold.

**Compound**	**AK1^T (current study)**	***T. subterraneus* C55^{T} [a]**
C16:0	**62.4**	**72.8**
C16:1 ω7c	**14.8**	**23.1**
C17:0	1.0	-
C17:1 Δ	**15.7**	-
C18:0	3.9	2.3
Iso-C18:0	-	1.3
C18:1	-	0.4
Unidentified (C16:0 10-methoxy)	1.7	-

[a]Hirayama, H.; Takai, K.; Inagaki, F.; Nealson, K.H.; Horikoshi, K. *Thiobacter subterraneus* gen. nov., sp. nov., an obligately chemolithoautotrophic, thermophilic, sulfur-oxidizing bacterium from a subsurface hot aquifer. *Int. J. Syst. Evol. Microbiol.* **2005**, *55*, 467–472.

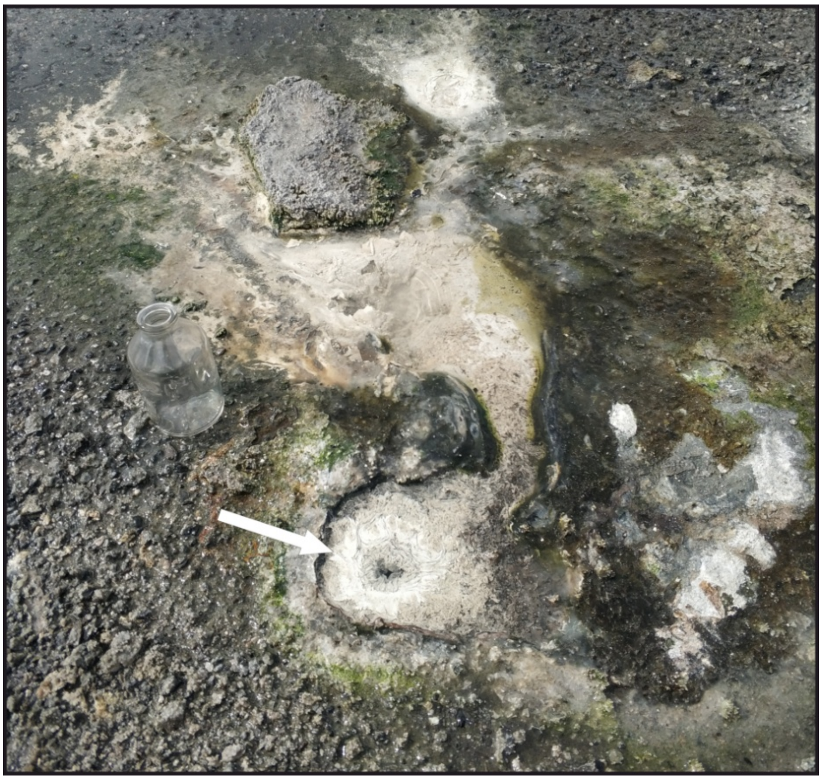

Supplementary Figure S1. Sampling site 4212 which is the isolation source of the strain AK1^T

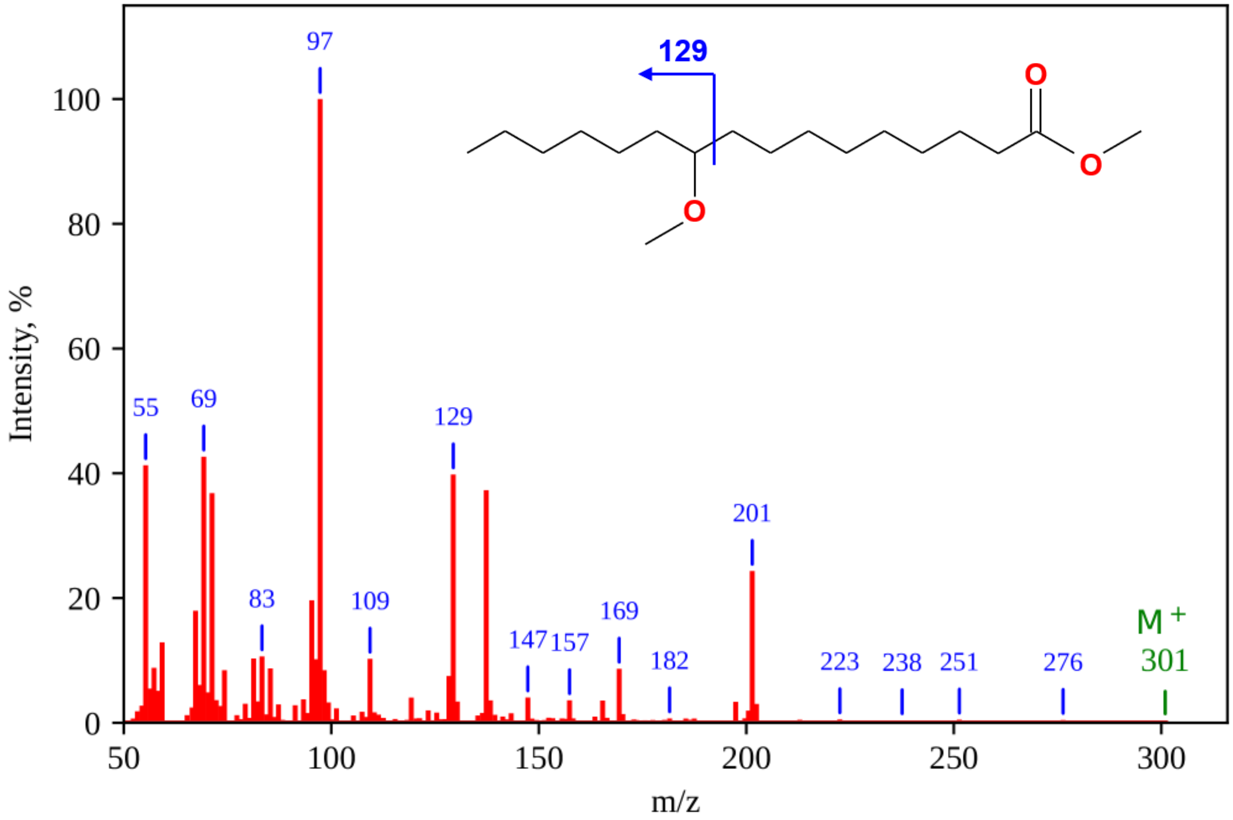

Supplementary Figure S2. Mass spectrum of the unidentified fatty acid methyl ester with an ECL (CP-Sil 88) = 19.22. ECL, equivalent chain length for the Agilent CP-Sil 88 column (highly polar cyanopropyl phase). According to the search in the NIST MS Search database, the fatty acid is probably methoxy substituted. The peak at m/z=301 could be either a molecular ion or a molecular ion without an easily cleavable fragment. The polar methoxy substituent increases retention time on a highly polar column, thus increasing ECL value similarly to, e.g., hydroxy-substituted fatty acids. The tentative structure is C16:0 10-methoxy (methyl 10-methoxyhexadecanoate).

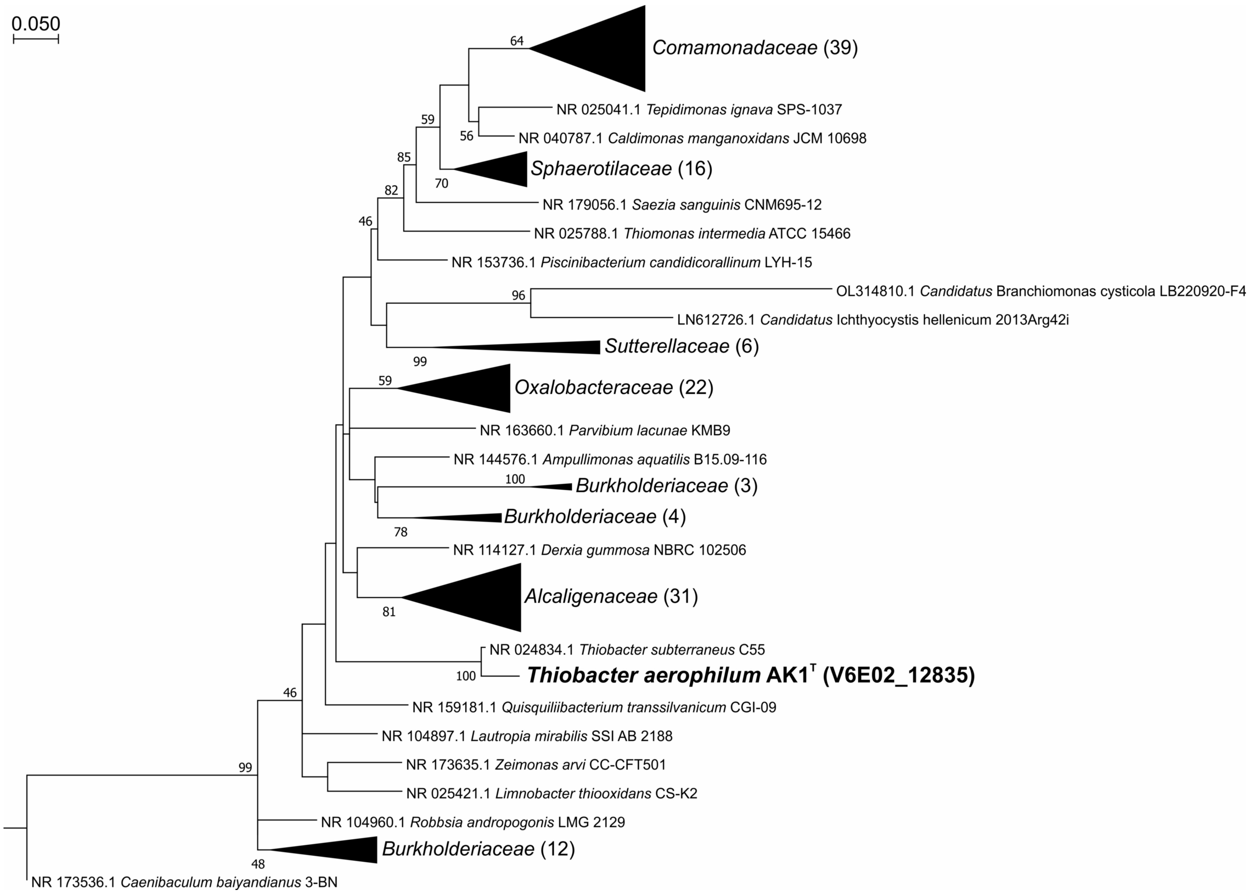

Supplementary Figure S3. A phylogenetic tree based on a comparison of 16S rRNA gene sequences, constructed by the maximum likelihood method, showing the position of the AK1^T strain (highlighted in bold) within the *Burkholderiales* order. The length of the branches corresponds to the number of substitutions per position, as amended taking into account the selected model (GTR, G + I, 4 categories). All positions with less than 95% coverage were excluded. The numbers in the nodes reflect the percentage of the bootstrap test (1000 repetitions), values less than 40% are not shown. *Escherichia coli* NBRC 102203 was used as an external group.

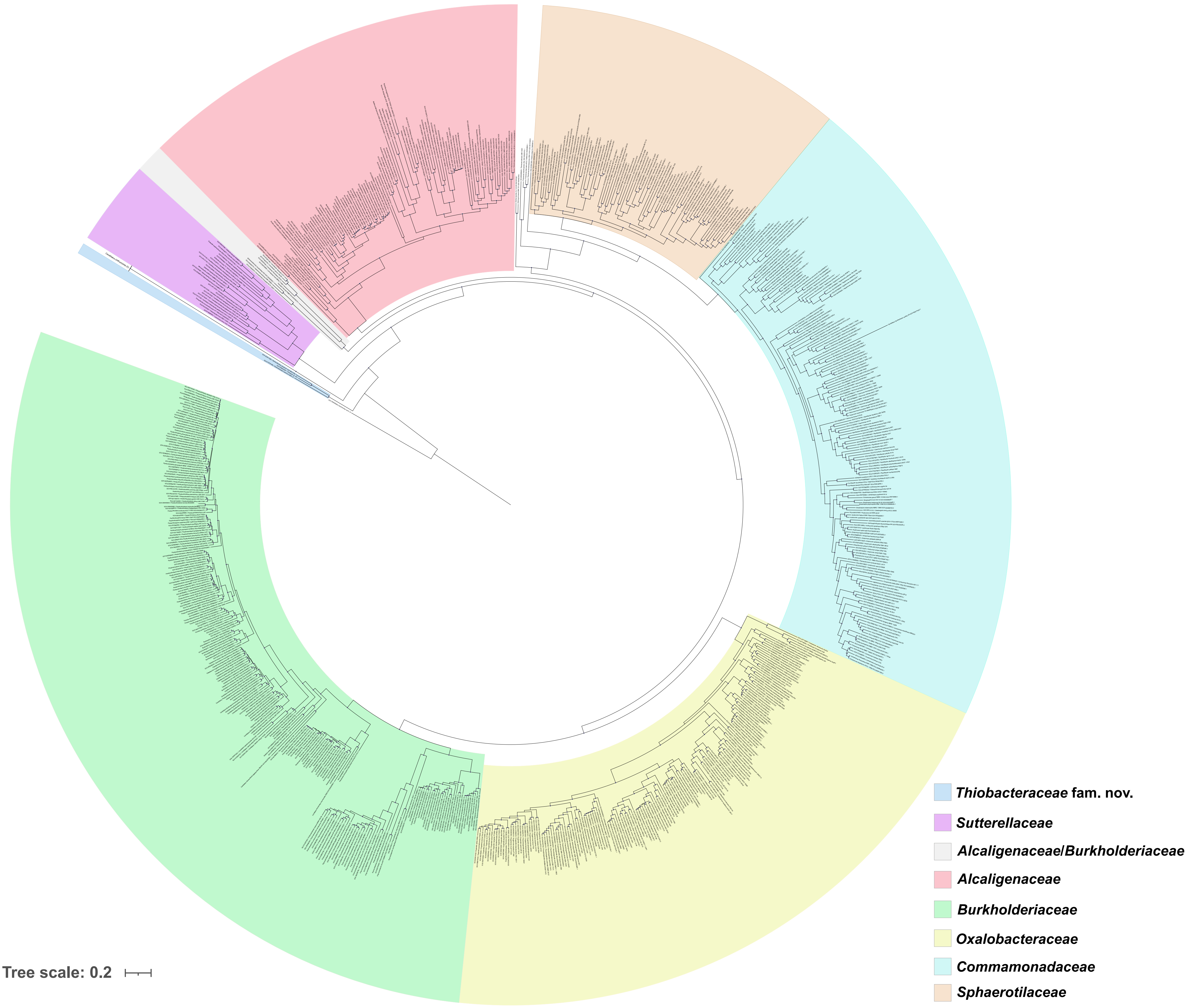

Supplementary Figure S4. Maximum likelihood phylogenetic tree based on comparison of 120 conserved proteins and showing the position of the strain AK1^{T} within the order *Burkholderiales*. The branch lengths correspond to the number of substitutions per site (see scale) according to the corrections associated with the LG+I+G4 model. The black circles at nodes indicate that percentages of corresponding support values are higher then 75%. *Escherichia coli* K-12 was as an outgroup.
